# The clinical classification of patients with acute heart failure at emergency department and its relation with management and outcome: a cross sectional study from Syria

**DOI:** 10.1186/s12872-025-04644-5

**Published:** 2025-03-18

**Authors:** Mohammad Aldli, Mohammad Alsultan, MhdAmin Alkhatib

**Affiliations:** 1 Department of cardiology, Damascus University- Faculty of Medicine, Damascus, Syria; 2 Department of Nephrology, Damascus University- Faculty of Medicine, Omar Ibn Abdulaziz Street, Al Mazah, Damascus, Syria

**Keywords:** Acute heart failure (AHF), Congestion, Outcomes, Right heart failure (RHF), Lung ultrasound (LUS), Echocardiography

## Abstract

**Introduction:**

To compare the clinical characteristics and outcomes of patients with acute heart failure (AHF) according to the 2016 European Society of Cardiology (ESC) guidelines taking into account isolated right HF (RHF) with left HF (LHF) phenotypes. Volume status was assessed by the clinical manifestations and lung ultrasound (LUS). The secondary aim was to study the role of echocardiography in congestion based on LUS and their relations with outcomes.

**Methods:**

This study included AHF patients, who referred to the emergency department (ED) at AL-Mouwasat and AL-Assad University Hospitals in Syria between May and August 2024. The same cardiologist reviewed medical reports, signs/ symptoms of decompensation, echocardiographic assessment, diagnosis, and treatment therapies.

**Results:**

Of 100 patients, 10 patients (10%) had isolated RHF and 90 patients (90%) had LHF, including warm-wet (n = 65, 65%), followed by cold-wet (n = 13, 13%), warm-dry (n = 10, 10%), and cold-dry (n = 2, 2%). Most discharged patients without admission were Warm-dry, meanwhile most of patients with cold-wet (76.9%) were admitted to intensive care unit (ICU). The longest in-hospital stays were in cold-wet (11.9 days) followed by isolated RHF (7.5 days). While in-hospital mortality was mainly in cold-wet (38.5%) followed by isolated RHF (20%). Diuretics dose was highest in cold-wet followed by isolated RHF, while hydration was predominantly in cold-wet. Using vasopressors and inotropes were predominantly in cold-wet. Systolic blood pressure (SBP), hemoglobin (Hb), sodium (Na), proximal right ventricular outflow tract (RVOT1), left ventricular end-diastolic internal diameter (LVIDd), Tricuspid annular systolic plane excursion (TAPSE), and systolic pulmonary atrial pressure (SPAP) correlated with hospital stays, while only SBP and Cr correlated with in-hospital mortality. The cut-off values of E/e’ ratio, isovolumic relaxation time (IVRT), and deceleration time (DT) were (12.5, 55ms, and 131.5 ms; respectively) and could predict congestion (guided by LUS) with sensitivities of (96%, 74%, and 62%; respectively) and specificities of (53%, 92%, and 84%; respectively).

**Conclusion:**

Classifying AHF patients into these five groups, based on clinical examination supporting by echocardiography and LUS evaluation can give better assessment of the AHF phenotypes and gives more details for management. The bedside diagnostic assessment by LUS and echocardiography is an easy tool and seems to be of great benefit in detecting congestion that enhances the treatment protocols.

## Introduction

Heart failure (HF) remains the leading cause of morbidity and mortality affecting about 1–2% of the population worldwide [1, 2]. HF is a complex clinical syndrome resulting from structural and functional impairment of ventricular filling or ejection of the blood, characterized by a constellation of symptoms (dyspnea, orthopnea, and lower limb swelling) and signs (elevated jugular venous pressure and pulmonary congestion) [3]. Hospitalizations for HF in the United States has been decreased until 2012; however, from 2013 to 2017, an increase in HF hospitalizations was observed. In 2017, there were 1.2 million HF hospitalizations in the United States among 924 000 patients with HF. This represents a 26% increase in HF hospitalizations and number of patients hospitalized with HF [1]. Patients with HF are classified with symptom and the stage of the disease. The American College of Cardiology /American Heart Association (ACC/AHA) HF staging approach emphasizes the importance of development and progression of disease, whereas the New York Heart Association (NYHA) functional classification focuses more on exercise tolerance in those with established HF [1].

Acute heart failure (AHF) is increasingly recognized as a distinct disorder with unique epidemiology, pathophysiology, treatments, and outcomes. The new therapeutic approaches have systematically failed to improve survival of patients with AHF [4]. The most recent European Society of Cardiology (ESC) guidelines have now changed from the previous clinical classification based on six phenotypic forms (worsening or decompensated chronic HF, pulmonary edema, hypertensive HF, cardiogenic shock, isolated right HF, and acute coronary syndrome and HF) to the new classification based on the intensity of congestion and perfusion [4]. The assessment of clinical phenotype based on peripheral perfusion (whereby normal perfusion is considered ’warm’ and symptoms or signs of hypoperfusion are considered ’cold’) and /or systemic congestion (whereby no congestion is considered ’dry’ and the presence of congestion is considered ’wet’). Given these phenotypes allow to classify patients into the following forms: warm-wet (well perfused and congested), cold-wet (hypoperfused and congested), cold-dry (hypoperfused without congestion), and warm-dry (compensated, well perfused without congestion) [4, 5]. This clinical classification has an important role on management and outcomes for patients with AHF [4, 5].

In addition to clinical evaluation, ultrasound (US) plays a considerable role in assessment of dyspnea for patients at emergency department. Moreover, assessment of filling pressures and congestion in HF depending on algorithms integrate mitral pulsed-wave inflow velocities (Mitral E and A wave velocity), pulsed-wave tissue Doppler imaging (TDI) e′ velocity, E/e’ ratio, peak velocity of the tricuspid regurgitation (TR) jet, and left atrium maximum volume index [6–8]. When these measures combined with lung ultrasound (LUS), this becomes a powerful bedside tool, that allow the diagnosis optimization of patients with dyspnea at emergency department and detect even the low degrees of pulmonary congestion [9].

Since the publication of the 2016 ESC guidelines, this classification has been assessed in a few studies, which described demographic, clinical, therapeutic approach, and outcomes for AHF- patients [4, 5, 10]. However, these studies did not take isolated right HF into account with left HF classifications. Also, these studies evaluated the congestion by clinical assessment without LUS evaluation [4, 5, 10]. Previous studies showed that LUS demonstrated a rapid diagnostic test to detect the congestion in AHF-patients with a high sensitivity (94–97%) and specificity (97%), that superior to clinical assessment and the chest X-ray, even with superimposed pneumonia [9]. Taking these considerations into account, we evaluated demographic, clinical, therapeutic approach, and outcomes of AHF- patients by classifying into four groups according to the 2016 ESC Guidelines with an additional group of isolated right HF. The assessment of volume status based on the clinical manifestations combined with LUS. The primary outcomes of this study were in-hospital mortality, admission days, and therapeutic managements. The secondary aim was to study the role of echocardiography in evaluating the congestion based on LUS positivity in AHF- patients and their relations with outcomes.

## Materials and methods

### Patients and data

This cross- sectional study included patients, who referred to the emergency departments (ED) at AL-Mouwasat and AL-Assad University Hospitals in Syria in the period, that extending between May and August 2024. The study protocol was approved by the Research Ethics Committee of Damascus University (Ethical Committee *N* = 3613, Damascus, Syria in September 2023) and by the Declaration of Helsinki. All patients obtained and received a copy of a written informed consent for publishing their clinical details. Patient confidentiality was maintained by inserting patients as a number without names on the worksheet program.

The inclusion criteria included all patients with symptoms and signs of acute decompensated heart failure (AHF). The exclusion criteria include; patients younger than 18 years, acute myocardial infarction, acute pulmonary embolism, patients with hospitalization for other reasons like sepsis and chronic obstructive pulmonary disease (COPD), and patients didn’t evaluate within 12 h after admission.

The study form was filled out by ED doctors within 12 h of hospital admission. Thereafter, the same cardiologist reviewed medical reports, signs and symptoms of decompensation, and echocardiography to make the final diagnosis. Data included age, gender, body mass index (BMI) [11], and body surface area (BSA) [12]. The information related to personal, medical and medication histories were also included [6]. Clinical signs and symptoms of AHF were evaluated according to the Framingham clinical criteria and ESC recommendations [6, 13]. Vital signs and laboratory tests were collected at the first time after ED referral. The Electrocardiogram (ECG) and rhythm (sinus rhythm or atrial fibrillation AF) were documented according to ESC recommendations [6].

### Echocardiography assessment

Echocardiography evaluation was performed by using GE vivid 8 and 9 devices within 12 hours of admission. The M-Mode method in Para Sternal Long Axis View (PLAX) and Para Sternal Short Axis View (PSAX) were relied upon to evaluate left ventricular (LV) dimensions, wall thickness, and ejection fraction (EF), as it is the fastest method [14]. Right ventricular (RV) dysfunction was evaluated based on: Tricuspid Annular Systolic Plane Excursion (TAPSE), and proximal RV outflow tract (RVOT1 in cm) by short axis view [15]. Diastolic dysfunction and left ventricular (LV) filling pressures, and were assessed by calculating: Mitral E wave velocity (m/sec) (E-wave velocity reflects the LA-LV pressure gradient during early diastole), which was assessed by Apical four-chamber with color flow imaging for optimal alignment of PW Doppler with blood flow. Pulsed-wave TDI e′ velocity (m/sec) lateral and septal e’ Apical four-chamber view: PW Doppler sample volume (usually 5–10 mm axial size) at lateral and septal basal regions so average e′ velocity can be computed. Average E/e’: average MV E velocity divided by mitral and septal annular e′ velocity. Mitral E-velocity Deceleration Time (DT): Time (ms) interval from peak E-wave along the slope of LV filling extrapolated to the zero-velocity baseline assessed by Apical four-chamber, pulsed Doppler sample volume between mitral leaflet tips. Isovolumic Relaxation Time IVRT (ms): five-chamber view, using Continuous Wave (CW) or Pulse Wave (PW) Doppler and placing sample volume in LV outflow tract to simultaneously display end of aortic ejection and onset of mitral inflow.

Left atria (LA) maximum volume index (mL/m^2^): Apical four- and two-chamber. The Tricuspid Regurgitation (TR) jet peak velocity (m/sec) was assessed by parasternal short axis and apical four-chamber view. The TR jet peak velocity > 2.8 m/sec supports the presence of elevated LV filling pressures.

The four recommended variables for identifying diastolic dysfunction and their abnormal cutoff values are annular e′ velocity: septal e′< 7 cm/sec, lateral e′< 10 cm/sec, average E/e′ ratio > 14, LA volume index > 34 mL/m^2^, and peak TR velocity > 2.8 m/Sect. [8]. LV diastolic function is normal if more than half of the available variables do not meet the cutoff values for identifying abnormal function. Meanwhile, LV diastolic dysfunction is present if more than half of the available parameters meet these cutoff values [16]. Systolic pulmonary artery pressure (SPAP) calculated by the pressure gradient between the right ventricular and atrium [17].

Lung ultrasound (LUS) was obtained by cardiac probe on the anterior and lateral chest, from the second to the fourth (on the right side to the fifth) intercostal spaces, and from the parasternal line to the axillary line [18]. The transducer should be placed in the intercostal space either perpendicular (longitudinal and sagittal) or in parallel orientation (transverse) to the ribs. Imaging depth depends on the size of the patient, but is usually set at ∼15–18 cm [9]. LUS was evaluated for assessment of pleural effusion and pulmonary congestion by studying B-lines and evaluating severity of congestion based on the number of lines. We used 8 zones protocol and quantified the congestion degree as follows; score 0 (no congestion B-lines < 5), score 1 (mild; B-lines between 5 and 15), score 2 (moderate; B-lines between 15 and 30), and score 3 (severe; B-lines > 30) [18].

After clinical and US evaluation (cardiac and lung), patients were classified into four groups based on congestion and perfusion according to ESC recommendations: no congestion with no hypoperfusion (dry-warm), congestion without hypoperfusion (wet-warm), hypoperfusion without congestion (dry-cold), and congestion with hypoperfusion (wet-cold). To categorize as congestion, at least one of clinical or LUS signs should be present: pulmonary crackles, peripheral edema, jugular congestion, hepatomegaly, ascites, LUS score > 0. Hypoperfusion was defined by the presence at least one of following: dizziness, cold extremities, mental confusion, or delayed capillary refill time. In addition to a fifth group of isolated right heart failure (RHF) after evaluation by echocardiography and exclude of left ventricular systolic or diastolic dysfunction according to ESC recommendations [19].

### Management and outcomes

Patients were assessed in the emergency office, where the study groups were divided into two calcifications; once managed in emergency and discharged without admission to refer to the cardiac clinic, while the second group was admitted to the hospital. The admitted patients were classified into three departments (ED, cardiology department, and ICU). Hemodynamically unstable patients (such as cardiogenic shock) were admitted to the ICU, meanwhile, hemodynamically stable patients were admitted to the cardiology department, and the status between them was admitted to the ED department until they improved and transmitted to the cardiology department to complete the study and treatments.

The primary outcomes include in-hospital death and stay (days of admission until discharged). Also, this included treatment protocols, that administered during hospitalization including; intravenous (IV) loop diuretics use and their total dose (total mg in all admission days), IV nitrates use, hydration with (IV normal saline -NS-), vasopressors use and days of administration (norepinephrine), and cardiac inotropes use and days of administration (dobutamine and dopamine). The relationship of echocardiographic features was also studied with primary outcomes (in-hospital death and stay). Furthermore, the measurements of the LV filling pressures including DT, IVRT, and E/e’ ratio were evaluated to detect congestion based on LUS features in this population.

### Statistical analysis

The data obtained has been inserted into Microsoft Excel worksheet and analysis by SPSS software version 23.0 (IBM, Armonk, New York, USA). The continuous data have been expressed as mean ± standard deviation (SD) and the comparison has been done using unpaired ‘t’ test. Categorical variables were compared by Chi square test. Studying correlations was by Pearson and Spearman tests. Also, we used Kruskal-Wallis H-test and Mann-Whitney U-Test to compare between independent variables. Receiver operating characteristic (ROC) curve was used to determine an appropriate cut-off for echocardiography measurements and calculating the sensitivity, specificity, and Area Under the Curve. A probability value (P. value ≤ 0.05) has been considered as statistically significant.

## Results

### Baseline characteristics (Table 1)

**Table 1:** Baseline characteristics

Variables				Isolated right (*n* = 10, 10%)	Left				Test	
					Cold-dry (*n* = 2, 2%)	Cold-wet (*n* = 13, 13%)	Warm-dry (*n* = 10, 10%)	Warm-wet (*n* = 65, 65%)	Test value	*P*. value
Age (Mean ± SD) (years)				57.8 ± 10.7	80.5 ± 6.4	66.2 ± 13	63.1 ± 6.8	63.7 ± 11.1	7.097^a^	0.131
Gender (n, %)	M			(4, 40%)	(2, 100%)	10 (7, 6.9%)	(3, 30%)	(34, 53.3%)	8.576^b^	0.073
	F			(6, 60%)	(0, 0%)	(3,23.1%)	(7, 70%)	(31, 47.7%)		
BMI (Mean ± SD) (kg/m^2^)				27.82 ± 8.1	23.5 ± 1.4	27.9 ± 6.7	31.28 ± 7.2	28.1 ± 5.7	4.768^a^	0.312
BSA (Mean ± SD) (m^2^)				1.9 ± 0.3	1.8 ± 0.1	2 ± 0.3	2.1 ± 0.3	2 ± 0.2	4.963^a^	0.291
***Medical History (n***,*** %)***										
Smoke				(5, 50%)	(1,50%)	(9, 69%)	(5, 50%)	(34, 52%)	1.471^b^	0.832
CHF				(7,70%)	(2,100%)	(10, 76.9%)	(4, 40%)	(49, 75.4%)	6.319^b^	0.177
HTN				(5, 50%)	(1, 50%)	(4, 30.8%)	(8, 80%)	(38, 58.5%)	6.255^b^	0.181
DM				(2, 20%)	(0, 0%)	(5, 38.5%)	(3, 30%)	(30, 46.2%)	5.330^b^	0.255
IHD				(0, 0%)	(2, 100%)	(6, 46.2%)	(5, 50%)	(36, 55.4%)	17.428^b^	0.002*
CVA				(0, 0%)	(0, 0%)	(1, 7.7%)	(1, 10%)	(5, 7.7%)	1.921^b^	0.750
CKD				(3, 30%)	(0, 0%)	(3, 23.1%)	(1, 10%)	(12, 18.5%)	2.299^b^	0.681
***Chronic treatments at home (n***,*** %)***										
ACEI				(0, 0%)	(1, 50%)	(0, 0%)	(1, 10%)	(10, 15.4%)	8.299^b^	0.081
ARBS				(1, 10%)	(0, 0%)	(0, 0%)	(1, 10%)	(6, 9.2%)	2.731^b^	0.604
*angiotensin receptor/neprilysin inhibitor*				(0, 0%)	(1, 50%)	(1, 7.7%)	(2, 20%)	(17, 26.2%)	8.254^b^	0.083
BB				(4, 40%)	(1, 50%)	(9, 69.2%)	(7, 70%)	(39, 60%)	2.612^b^	0.625
Loop diuretics				(7, 70%)	(2, 100%)	(10, 76.9%)	(4, 40%)	(43, 66.2%)	5.283^b^	0.259
MRA				(2, 20%)	(1, 50%)	(2, 15.4%)	(2, 20%)	(21, 32.3%)	2.848^b^	0.584
Digoxin				(1, 10%)	(0, 0%)	(3, 23.1%)	(2, 20%)	(10, 15.4%)	1.567^b^	0.815
SGLT2				(0, 0%)	(0, 0%)	(2, 15.4%)	(3, 30%)	(13, 20%)	5.847^b^	0.211
**Decompensation characteristics**										
***Clinical***										
SBP (Mean ± SD) (mmHg)				106.5 ± 28.2	77.5 ± 3.5	75.4 ± 16.4	153 ± 29.1	120.2 ± 26.9	42.545^a^	0.000*
Crackles (n, %)				(6, 60%)	(0, 0%)	(13, 100%)	(0, 0%)	(53, 81.5%)	42.949^b^	0.000*
Edema (n, %)				(10, 100%)	(0, 0%)	(12, 92.3%)	(0, 0%)	(51, 78.5%)	37.686^b^	0.000*
Plural effusion (n, %)				-----	(0, 0%)	(8, 61.5%)	(0, 0%)	(25, 38.5%)	14.349^b^	0.002*
AF (n, %)				(3, 30%)	(0, 0%)	(8, 61.5%)	(2, 20%)	(12, 18.5%)	10.737^b^	0.030*
NYHA (n, %)		1		(0, 0%)	(0, 0%)	(0, 0%)	(0, 0%)	(0, 0%)	36.431	0.000*
		2		(1, 10%)	(2, 100%)	(0, 0%)	(3, 30%)	(5, 7.7%)		
		3		(1, 10%)	(0, 0%)	(0, 0%)	(6, 60%)	(9, 13.8%)		
		4		(8, 80%)	(0, 0%)	(13, 100%)	(1, 10%)	(51, 78.5%)		
***Lab tests at admission*** (Mean ± SD)										
Hb (g/dL)				10.3 ± 3.1	12.6 ± 1.3	11.7 ± 2.8	12.5 ± 2	10.8 ± 2.1	8.344^a^	0.080
Cr (mg/dL)				1.7 ± 1.4	1.2 ± 0.1	2.4 ± 1.1	1.3 ± 0.4	1.6 ± 0.9	10.865^a^	0.028*
Na (mEq/L)				134.2 ± 3.7	130 ± 8.5	129.9 ± 5.6	137.4 ± 3.4	133.7 ± 5.2	11.3^a^	0.023*
***US at admission***										
LVIDd (Mean ± SD) (cm/m^2^)				-----	3.5 ± 0.4	3.4 ± 0.8	2.5 ± 0.4	2.9 ± 0.6	11.995^a^	0.007*
IVSd (Mean ± SD) (cm)				-----	1.1 ± 0	1 ± 0.2	1.4 ± 0.1	1.2 ± 0.3	9.786^a^	0.020*
LA. VoL (Mean ± SD) (ml/m^2^)				-----	44.5 ± 21.9	66.2 ± 33.9	46.1 ± 16.8	51.9 ± 22.8	3.609^a^	0.307
SPAP (Mean ± SD) (mmHg)				58.3 ± 22.1	20 ± 5.7	46.2 ± 11.2	33.8 ± 13.1	48.7 ± 13.2	13.381^a^	0.004*
TAPSE (Mean ± SD) (cm)				1.38 ± 0.48	1.6 ± 0.1	1.3 ± 0.4	1.9 ± 0.4	1.6 ± 0.5	9.421^a^	0.024*
RVOT1 (Mean ± SD) (cm)				4.23 ± 0.59	3.2 ± 0.6	4.1 ± 0.6	3.3 ± 0.4	3.6 ± 0.6	10.236^a^	0.017*
E/e’- Average (Mean ± SD)				-----	9.5 ± 3.5	21.9 ± 6.1	16.2 ± 8	21.4 ± 8.4	10.980^a^	0.012*
DT (Mean ± SD) (ms)				-----	226.5 ± 23.3	104.6 ± 25.4	170.5 ± 65.1	138 ± 56.7	13.683^a^	0.003*
IVRT (Mean ± SD) (ms)				-----	80 ± 18.4	44.2 ± 16.2	66.3 ± 16.4	48.1 ± 17.1	13.620^a^	0.003*
EF (Mean ± SD) (%)				-----	32 ± 11.3	24.3 ± 11.9	54.7 ± 8.8	42 ± 14.9	23.377^a^	0.000*
LUS Score (n, %)			0	--	(2, 100%)	(0, 0%)	(10, 100%)	(1, 1.5%)	75.187	0.000*
			1	--	(0, 0%)	(0, 0%)	(0, 0%)	(19, 29.2%)		
			2	--	(0, 0%)	(2, 15.4%)	(0, 0%)	(18, 27.7%)		
			3	--	(0, 0%)	(11, 84.6%)	(0, 0%)	(27, 41.5%)		
***Management and outcomes***										
Discharge without admission				(0, 0%)	(1, 50%)	(0, 0%)	(8, 80%)	(2, 3.1%)	38.660^b^	0.000*
Admission at (n, %)		Card. Dep		(1, 10%)	(1, 50%)	(0, 0%)	(0, 0%)	(7, 10.8%)	23.438	0.003*
		ED		(8, 80%)	(0, 0%)	(3, 23.1%)	(1, 10%)	(43, 66.2%)		
		ICU		(1, 10%)	(0, 0%)	(10, 76.9%)	(1, 10%)	(13, 20%)		
Admission days (Mean ± SD) (days)				7.5 ± 5.3	1 ± ----	11.9 ± 3.4	1.5 ± 0.7	5.4 ± 4.6	24.001^a^	0.000*
In-hospital mortality (n, %)				(2, 20%)	(0, 0%)	(5, 38.5%)	(0, 0%)	(1, 1.6%)	15.823^b^	0.000*
IV- Diuretics (mg/days) (Mean ± SD) (total mg/admission days)				1160 ± 1091	----	1596 ± 704.8	60 ± 54.2	664.6 ± 590	29.2^a^	0.000*
IV- Nitrate (n, %)				(0, 0%)	(0, 0%)	(0, 0%)	(2, 20%)	(16, 24.6%)	11.722^b^	0.020*
NS (n, %)				(2, 20%)	(2, 100%)	(7, 53.8%)	(0, 0%)	(5, 7.7%)	24.726^b^	0.000*
Noradrenaline		Use (n, %)		(4, 40%)	(0, 0%)	(12, 92.3%)	(0, 0%)	(3, 4.6%)	52,419^b^	0.000*
		Days (Mean ± SD)		6 ± 6.2	----	4.1 ± 2.4	----	4.3 ± 3.1	0.037^a^	0.982
Dopamine		Use (n, %)		(1, 10%)	(0, 0%)	(1, 7.7%)	(0, 0%)	(0, 0%)	6.055^b^	0.195
		Days (Mean ± SD)		1.5 ± 3	----	0.4 ± 1.4	----	0 ± 0	1.417^a^	0.492
Dobutamine		Use (n, %)		(2, 20%)	(0, 0%)	(11, 84.6%)	(0, 0%)	(7, 10.8%)	34.494^b^	0.000*
		Days (Mean ± SD)		4.8 ± 7.1	----	5.4 ± 4.1	----	8 ± 7.2	0.797	0.671
Inotropes combination (n, %)				(3, 30%)	(0, 0%)	(10, 76.9%)	(0, 0%)	(2, 3.1%)	40.416^b^	0.000*

BMI: body mass index, BSA: body surface area, CHF: chronic heart failure, HTN: hypertension, DM: diabetes mellitus, IHD: ischemic heart disease, CVA: cerebrovascular accident, CKD: chronic kidney disease, ACEI: Angiotensin-converting enzyme inhibitors, ARBS: Angiotensin receptor blockers, BB: beta-blockers, MRA: mineralocorticoid receptor antagonist, SGLT2: sodium/glucose cotransporter2 inhibitor, SBP: systolic blood pressure, AF: atrial fibrillation, NYHA: the New York Heart Association, Hb: hemoglobin, Cr: serum creatinine, Na: serum sodium, US: ultrasound, LVIDd: left ventricular end-diastolic internal diameter, IVSd: interventricular septum end-diastolic thickness, LA. VoL: left atrial maximum volume index, SPAP: systolic pulmonary atrial pressure, TAPSE: Tricuspid Annular Systolic Plane Excursion, RVOT1: proximal right ventricular outflow tract, E: mitral E wave velocity, e’: pulsed-wave TDI e′ velocity, DT: deceleration time, IVRT: isovolumic relaxation time, EF: ejection fraction, LUS: lung ultrasound, Card. Dep: cardiac department, ED: emergency department, ICU: intensive care units, IV: intravenous, NS: normal saline

Of 100 patients (Table 1), 10 patients (10%) had isolated right HF and 90 patients (90%) had left HF. The patients with left HF included warm-wet (*n* = 65, 65%), followed by cold-wet (*n* = 13, 13%), warm-dry (*n* = 10, 10%), and cold-dry (*n* = 2, 2%). Males comprised 53 patients (53%) and 47 patients (47%) were females. Patients with left HF were divided into clinical classifications as follows; 2 patients with cold-dry, 13 patients with cold-wet, 10 patients with warm-dry, and 65 patients with warm-wet. The mean age was 57.8 years in isolated right HF, meanwhile, the mean age was 80.5 years in cold-dry patients, 66.2 years in cold-wet patients, 63.1 years in warm-dry patients, and 63.7 years in warm-wet patients.

**Fig. 1:**
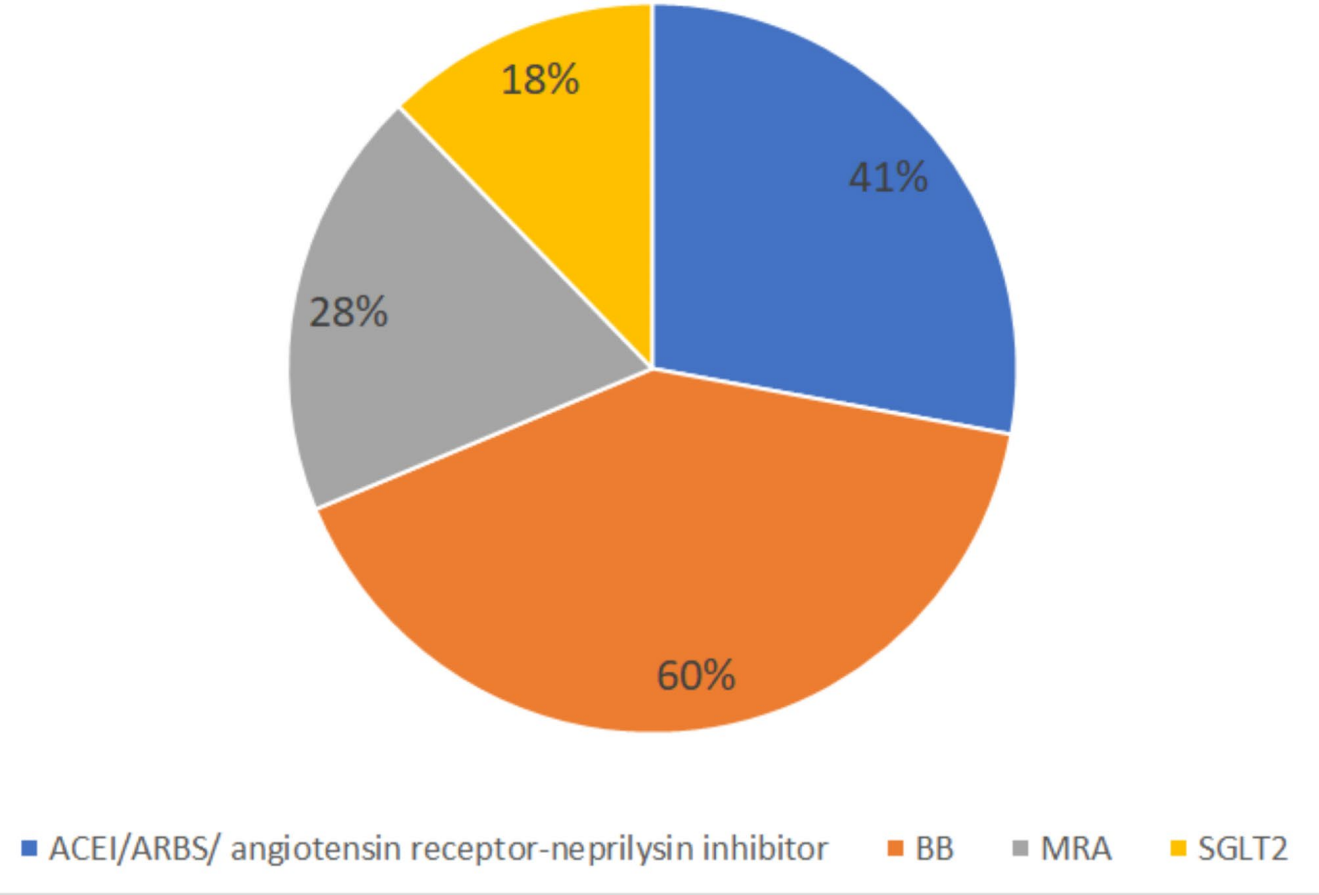
Guideline direct medical therapy (GDMT), including ACEI/ARBS/ angiotensin receptor-neprilysin inhibitor, BB, MRA, and SGLT2 inhibitors

The patients had a high number of co-morbidities, the most frequent being smoking history, CHF, HTN, IHD, and DM, where only IHD showed the difference between study groups (*P* = 0.002), (Table 1). With respect to the chronic treatments, 41% of patients received ACEI/ARBS/ angiotensin receptor-neprilysin inhibitor, 60% on BB, 66% on diuretics, and 28% on MRA. There were no differences in chronic treatments between groups (Table 1; Fig. 1).

All the clinical and lab characteristics showed differences between groups (Table 1). The mean SBP was lowest in the Cold-wet group (75.4 mmHg) and highest in the Warm-dry group (153 mmHg). Crackles were observed in 100% of the Cold-wet group followed by the Warm-wet group (81.5%). Of note, the isolated RHF group showed crackles in 60% of patients, however, these crackles did not belong to congestion after echocardiographic and LUS evaluations (like crackles in patients with pulmonary fibrosis). Edema showed in 100% of isolated RHF followed by 92.3% of the Cold-wet group. Also, the Cold-wet group showed the highest proportions of pleural effusion (61.5%), AF (61.5%), and NYHA type IV (100%) between study groups. Cr and Na showed differences between groups. Cr level was highest in the Cold-wet group (2.4 mg/dL), followed by the isolated RHF group (1.7 mg/dL), meanwhile, the lowest Na levels (129.9 mEq/L) were in the Cold-wet group.

**Fig. 2:**
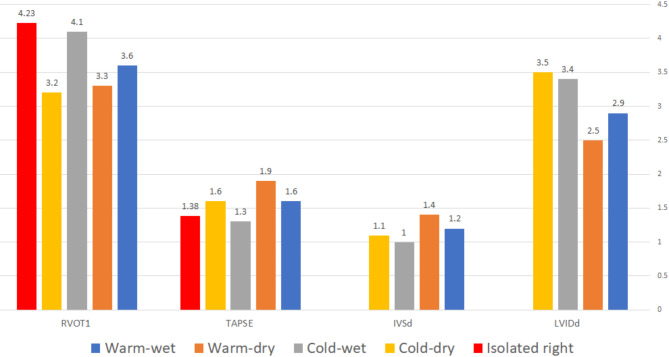
Cardiac ultrasound (US) characteristics at admission; shows the means of LVIDd, IVSD, TAPSE, RVOT1 between HF groups

**Fig. 3:**
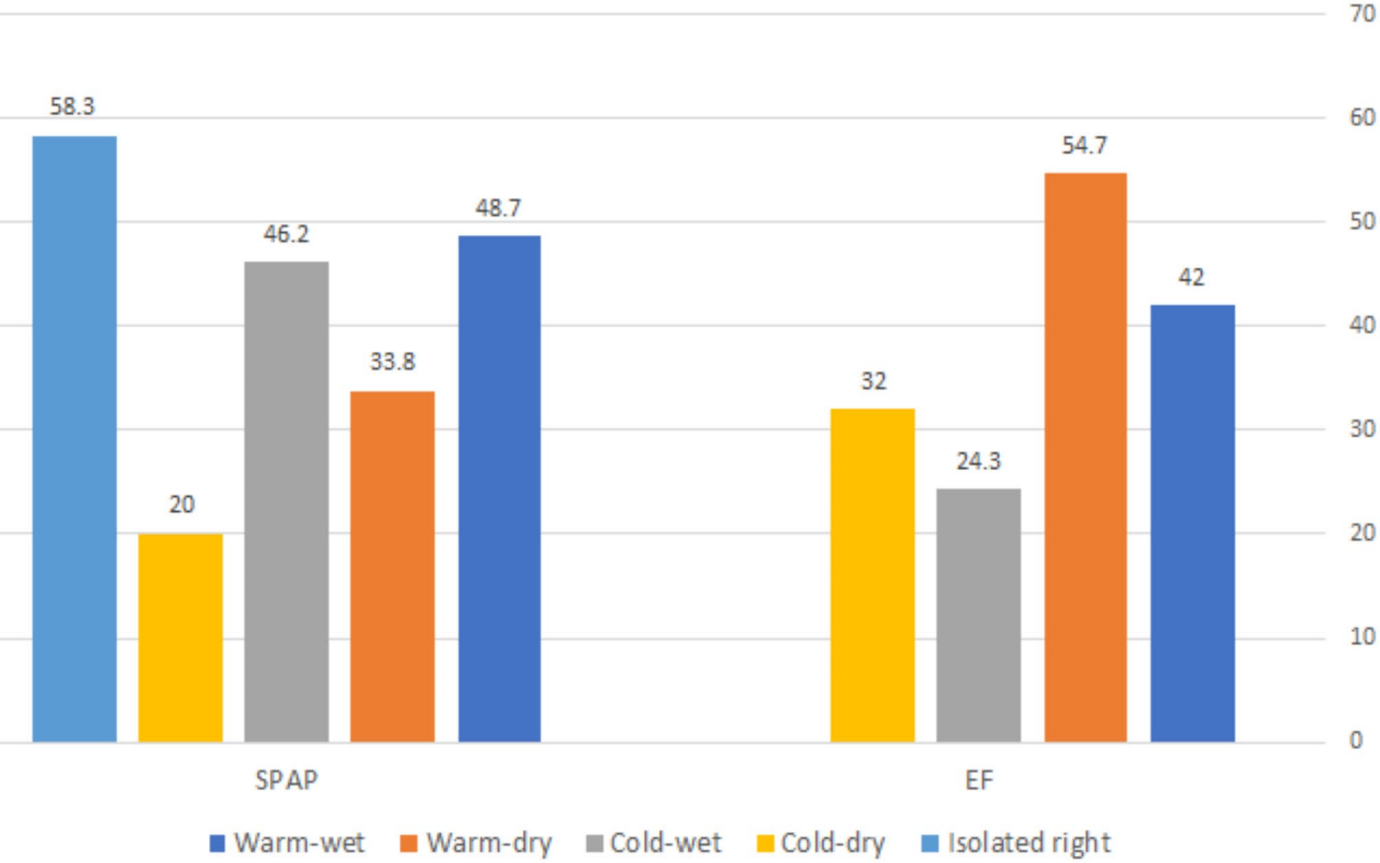
Cardiac US characteristics at admission; shows the means of EF and SPAP between HF groups

All the US characteristics at admission showed significant differences between HF groups except LA-VoL (Table 1). The mean LVIDd (Fig. 2) was higher in the Cold-dry group (3.5 cm/ m^2^) and lowest in warm-dry (2.5 cm/m^2^). The highest mean of IVSd (Fig. 2) was in the Warm-dry group (1.4 cm) and lowest in the cold-wet group (1 cm). The Cold-wet group showed the highest levels of LA-VoL (66.2 mL/ m^2^) and the lowest level of TAPSE (1.3 cm) (Fig. 2). The isolated RHF group showed the highest levels of RVOT1 (4.23 cm) followed by the cold-wet group (4.1 cm) (Fig. 2). E/e’ Average was higher in the cold-wet group (21.9), while IVRT and DT were lower in the cold-wet group (104.6 ms and 44.2 ms; respectively). Meanwhile, the mean SPAP (Fig. 3) was higher in the isolated RHF group (58.3 mmHg) followed by the Warm-wet group (48.7 mmHg) and Cold-wet group (46.2 mmHg). The mean EF (Fig. 3) was lowest in the cold-wet group (24.3%) followed by the cold-dry group (32%), warm-wet group (42%), and warm-dry group (54.7%). LUS scores 1 and 2 were most common in the Warm-wet group (29.2% and 27.7%; respectively), meanwhile, the LUS score 3 was most common in the Cold-wet group (84.6%).

### Management and outcomes across clinical phenotypes (Table 1)

Patients were divided into two categories (Table 1). The first class was treated in the emergency office and discharged without admission; this class included 11 patients (*P* = 0.000), most of them in the Warm-dry group (*n* = 8). The second class was admitted to the hospital (*n* = 89 patients) into three departments (*P* = 0.003). Most patients in isolated RHF (80%) and warm-wet (66.2%) groups were admitted to the ED department, while most of the patients in the cold-wet group (76.9%) were admitted to the ICU. Admission days showed significant differences between groups (*P* = 0.000), where the longest admission days were observed in the cold-wet group (11.9 days), followed by the isolated right group (7.5 days). On the other hand, in-hospital mortality was reported in 8 patients (8% of the total sample) (*P* = 0.000), where 5 patients (5/13; 38.5%) were in the cold-wet group followed by the isolated RHF group (2 patients; 20%).

All treatments except dopamine showed significant differences between groups (Table 1). The mean dose of total IV diuretics during admission days (*P* = 0.000) was highest in the cold-wet group (1596 mg) followed by the isolated RHF group (1160 mg). Also, nitrate and hydration (NS) showed differences between groups (*P* = 0.020 and 0.000; respectively), where nitrate was predominantly in the warm-wet group, while NS was predominantly in the cold-wet group. Using noradrenaline, dobutamine, and inotropes combination showed significant differences between groups (*P* = 0.000 for each), where their predominant use was in the cold-wet group.

### Relations of clinical, labs, and US with outcomes (Table 2)

**Table 2:** Relations of clinical, labs, and US with outcomes

Variables		Hospital stays				Variables		Test	Hospital Mortality			*P*. value
		Test		Value	*P*				Death	Discharge	Value	
Age		Spearman Correlation		-0.181	0.107	Age		Mann- Whitney *U-*test **Median**	63.5	64	291	0.713
BMI				0.062	0.580	BMI			25.1	27.3	240	0.264
SBP				-0.240	0.031*	SBP			77.5	110	86.5	0.001*
Hb				-0.287	0.009*	HB			12.5	11	247	0.311
Cr				0.144	0.201	Cr			1.85	1.3	156	0.019*
Na				-0.305	0.001*	Na			130	134	188	0.059
IVSd				-0.154	0.195	IVSd			0.95	1.1	145	0.194
RVOT				0.431	0.000*	RVOT			3.65	3.6	211.5	0.977
TAPSE				-0.248	0.035*	TAPSE			1.3	1.5	156	0.277
LVIDd				0.331	0.004*	LVIDd			3.07	3	194	0.718
SPAP				0.303	0.009*	SPAP			46	48	203	0.849
Edema	1	Kruskal-Wallis H-test **Median**	3.5	14.870	0.002*	Edema	1	Chi- Square **(%)**	7.7%	92.3%	3.306	0.347
	2		4				2		3.8%	96.2%		
	3		7				3		16.7%	83.3%		
	4		9									
							4		20%	80%		
AF	No	Mann-Whitney *U-*test **Median**	4	377	0.010*	AF	No		62.5%	75.3%	0.554	0.456
	Yes		8				Yes		37.5%	24.6%		

BMI: body mass index, SBP: systolic blood pressure, Hb: hemoglobin, Cr: serum creatinine, Na: serum sodium, IVSd: interventricular septum end-diastolic thickness, TAPSE: Tricuspid Annular Systolic Plane Excursion, RVOT1: proximal right ventricular outflow tract, LVIDd: left ventricular end-diastolic internal diameter, SPAP: systolic pulmonary atrial pressure, AF: atrial fibrillation

Systolic blood pressure (SBP), hemoglobin (Hb), and sodium (Na) showed negative correlations with hospital stay (*P* = 0.031, 0.009, and 0.001; respectively). RVOT1, LVIDd, and SPAP showed positive correlations with hospital stay (*P* = 0.000, 0.004, and 0.009; respectively); however, TAPSE showed a negative correlation (*P* = 0.035). Edema showed a positive correlation with hospital stay (*P* = 0.002), where the admission days increased with an increase in the grade of edema. Also, AF correlated with hospital stay (*P* = 0.010), where patients with AF showed longer admission days than those without AF (8 vs. 4 days; respectively).

On the other hand, only SBP and Cr showed correlations with in-hospital mortality (death/discharge). SBP was higher (110 mmHg) in discharged patients compared with death patients (77.5 mmHg) (*P* = 0.001), and Cr was higher in death patients (1.85 mg/dL) compared with discharged patients (1.3 mg/dL) (*P* = 0.019).

### Cardiac US of the LHF to detect congestion guided by LUS (Table 3)

**Table 3:** Correlations of left HF cardiac US measurements with LUS score (Kruskal-Wallis H-test), and ROC analysis for cardiac US measurements to predict congestion by LUS scores

Kruskal-Wallis H-test					ROC analysis				
Variables	LUS Score	Median	H-test value	*P*-value	Variables	LUS score		95%CI for AUC	*P*-value
**IVRT**	**0**	67	19.676	0.000*	**IVRT**	**Cutoff**	55	0.732–0.938	0.000
	**1**	53				**Sensitivity**	0.74		
	**2**	48				**Specificity**	0.92		
	**3**	39.5				**AUC**	0.835		
**E/e’ average**	**0**	12	18.701	0.000*	**E/e’ average**	**Cutoff**	12.5	0.602–0.941	0.002
	**1**	16				**Sensitivity**	0.96		
	**2**	18.5				**Specificity**	0.53		
	**3**	21.5				**AUC**	0.771		
**DT**	**0**	167	25.16	0.000*	**DT**	**Cutoff**	131.5	0.631–0.911	0.002
	**1**	150				**Sensitivity**	0.62		
	**2**	132				**Specificity**	0.84		
	**3**	112				**AUC**	0.771		

LUS: lung ultrasound, IVRT: isovolumic relaxation time, E: mitral E wave velocity, e’: pulsed-wave TDI e′ velocity, DT: deceleration time

**Fig. 4:**
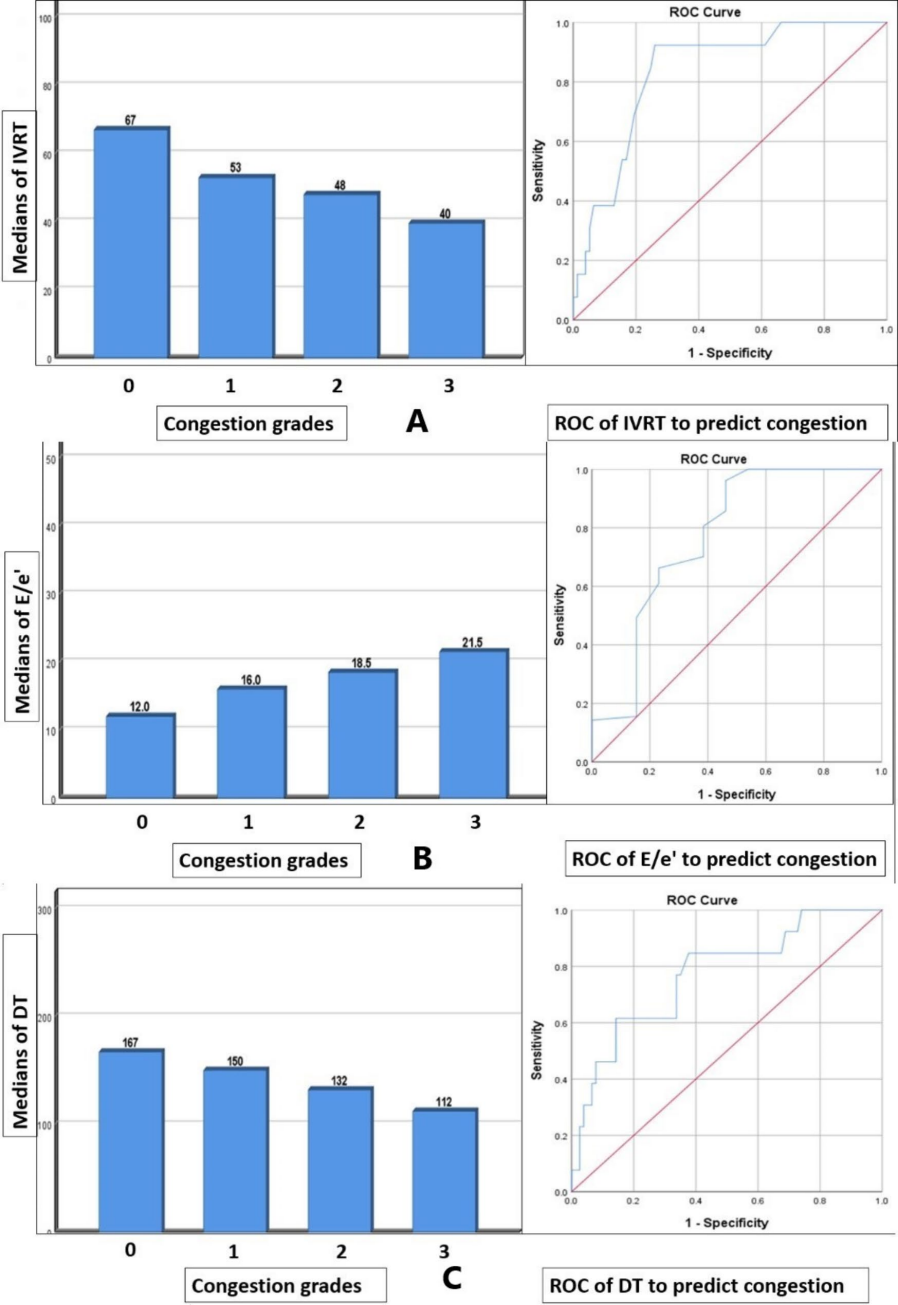
Correlations of cardiac US with LUS score, and the ROC analysis of cardiac US to predict congestion: **(A)**: IVRT values decreased with increased LUS score, and the ROC analysis to predict congestion. **(B)**: E/e’ average values increased with increased LUS score, and the ROC analysis to predict congestion. **(C)**: DT values decreased with increased LUS score, and the ROC analysis to predict congestion

IVRT, E/e’ average, and DT showed significant differences between scores of LUS (*P* = 0.000 for each) (Table 3, Fig. 4). IVRT values (Fig. 4) decreased with increased LUS score, where the lowest IVRT value (39.5 ms) was shown in LUS score 3. E/e’ average values (Fig. 4) increased with increased LUS score, where the highest E/e’ average value (21.5) showed in LUS score 3. DT values (Fig. 4) decreased with increased LUS score, where the lowest DT value (112 ms) showed in LUS score 3.

On the other hand, the IVRT cut-off value (55ms) could predict congestion (guided by LUS) with sensitivity (74%) and specificity (92%), (AUC = 0.835; *P* = 0.000; CI = 0.732–0.938) (Fig. 4). E/e’ average cut-off value (12.5) could predict congestion with sensitivity (96%) and specificity (53%), (AUC = 0.771; *P* = 0.002; CI = 0.602–0.941) (Fig. 4). DT cut-off value (131.5ms) could predict congestion with sensitivity (62%) and specificity (84%), (AUC = 0.771; *P* = 0.002; CI = 0.631–0.911) (Fig. 4).

## Discussion

This study compared patients with AHF according to the classification of the 2016 ESC guidelines [19], based on clinical examination and US obtained at bedside to detect four distinct phenotypes of left HF with additional isolated RHF phenotype. Furthermore, the baseline characteristics, in-hospital therapies, and outcomes were compared between these five groups. The main phenotype in this study was warm-wet group (*n* = 65, 65%), while the cold-dry group (*n* = 2, 2%) presented the lowest distribution. In line with studies by Chioncel et al. [10], Javaloyes et al. [4], and Palazzuoli et al. [20] were warm-wet phenotype reported in 69.9%, 76%, and 73.3% respectively. Also, the lower phenotype group were cold- dry in studies by Chioncel et al. [10] and Javaloyes et al. [4] where the cold- dry presented in 0.4% and 0.9%, respectively. Meanwhile Palazzuoli et al. [20] found that warm-dry group (5%) was the lowest group of AHF. Only study by Javaloyes et al. [4] reported the patients who discharged directly from the ED without admission, where the mainly phenotype of those patients was warm-wet phenotype [4]. Our study reported that 11 patients discharged without admission, where most of them in Warm-dry group. Also, this study reported the admission into three departments, where the ED admissions showed predominantly isolated right HF (80%) and warm-wet (66.2%) groups, while ICU admissions showed predominantly cold-wet group (76.9%).

The primary outcomes of this study were in-hospital mortality, in-hospital stays, and therapeutic managements. In-hospital mortality was observed in 8 patients (8% of the total sample), where 5 patients (38.5%) in cold-wet group followed by isolated right group and the longest days of admission also showed in cold-wet group (11.9 days) followed by isolated right group (7.5 days). Meanwhile, the highest doses of the total IV diuretics were in cold-wet group (1596 mg) followed by isolated right group (1160 mg) and use of NS (hydration) was predominantly in cold-wet group, in addition those groups had the highest serum Cr levels. Moreover, inotropes and vasopressors use (noradrenaline, dobutamine, and inotropes combination) were predominantly in cold-wet group.

Similarly, Chioncel et al. [10] found in-hospital mortality in 5.3% of the population, where the highest mortality rates, one-year all-cause mortality rates, and longer hospital stay were also higher in cold-wet patients. Also, in respect to previous classification of ESC 2008, a previous study by Chioncel et al. [5] showed that the highest mortality rates were noted in congestion and hypoperfusion patients. Meanwhile, Javaloyes et al. [4] found that hypoperfusion (cold) groups had higher in-hospital and 1-year mortality that did not relate to basal profile or co-morbidities. Palazzuoli et al. [20] demonstrated 23 deaths at 30 days follow-up, where cold-wet patients showed higher mortality and cold groups showed a worse outcome compared to the warm groups. Also, congestion (wet) groups with or without hypoperfusion showed longer hospital stay and more frequent use of IV diuretics compared with other groups [4]. Additionally, inotropes and vasopressors were used more common in the cold-wet group [10].

This increase in mortality is mainly explained by a lower mean BP and the inverse relation of BP with mortality in patients with AHF, while longer hospital stays perhaps due to prolong in-hospital treatment of congestion with diuretics and vasopressors [5, 21]. This hypoperfusion state was mainly correlated with in-hospital and 1-year mortality in previous studies [4, 10].

In respect to congestion, previous studies found that congestion did not affect in-hospital mortality [4, 10, 22], meanwhile Chioncel et al. [10] found that patients with congestion at discharge had a significantly higher 1-year mortality. Similarly, our study found a correlation between edema (congestion) and hospital stay, however, it did not correlate with in-hospital mortality (Table 2). This study also highlighted the correlation between SBP and outcomes (Table 2), where SBP showed a negative correlation with in-hospital stays and higher levels of SBP was observed in discharged patients compared with death patients (in-hospital mortality). On the other hand, this study showed that isolated right HF was the second group in mortality, hospital stay, and diuretics use. This mainly explained by congestion signs of isolated right HF patients, where they showed a high proportion of edema (100%). Of note, the two mortalities in isolated right HF group (representing 20%) showed hypoperfusion, which included under the cold-wet phenotype. When isolated RHF has several etiologies, different managements, and prognoses, that differ from LHF, it is rational to study RHF as an isolated group compared to LHF groups. Also, isolated RHF has no robust chronic therapies or recommendations for treatment, so this group should be studied as an isolated group in future large control trials. It is well-known that isolated RHF patients have an obvious congestion (wet) with either low or normal BP (warm or dry), so the RHF patients could classified under wet-warm or -dry phenotype.

Concerning guideline direct medical therapy (GDMT), which is recommended in patients with HF, it showed a reduction in hospitalization and mortality [6]. We found that 41% of our HF patients received ACEI/ARBS/ angiotensin receptor-neprilysin inhibitor, 60% were received BB, and 28% were received MRA. Meanwhile, only 18% of our patients received SGLT2 inhibitors. This observation of low GDMT prescriptions obligates more attention and an attempt for more GDMT prescriptions in our HF patients.

Furthermore, SGLT2 inhibitors were extensively studied in patients with RHF. Sarak et al. [23], found that SGLT2 inhibition had no impact on RV mass index in patients with T2DM, coronary artery disease and normal LVEF. Meanwhile, a meta-analysis by Tufan Cinar et al. [24], found favorable effects of SGLT2 inhibitors on RV function including TAPSE, SPAP, and fractional area change in HF patients. It seems that SGLT2 inhibitors have benefits in both RHF and LHF, however, the benefits in RHF need more approval.

Atrial fibrillation (AF) occurrence is common in AHF, where it can be a cause of AHF decompensation. The AATAC trial showed the superiority of catheter ablation (CA) over medical therapy in reducing AF recurrence, the primary endpoint, hospitalizations, and mortality [6]. The meta-analysis by Şaylık et al. for AF in HF patients showed that CA therapy is associated with greater improvements in LVEF and quality of life [25]. Also, according to ESC Guidelines 2024 for AF patients with HF showed that catheter ablation should be considered in selected AF patients with HFrEF to reduce HF hospitalization and prolong survival [26]. There is an increase in operator experience that facilitate the effectiveness of CA, however, arrhythmia recurrence is a common problem [27]. Pulmonary vein (PV) isolation (PVI) is the cornerstone of ablation in AF treatment [27]. The new technique of pulsed-field ablation (PFA), compared to the standard ablation methods with radiofrequency or cryoenergy, showed that the use of PFA for PVI in patients with paroxysmal and persistent AF has yielded favorable results [27].

In the current guidelines, echocardiography is recommended as an easily accessible and cost-effective tool in the diagnostic workup and prognosis of HF patients [28]. Right ventricular (RV) function is an important prognostic factor and patients with RV dysfunction have a poorer prognosis, which included an increase of cardiac death and hospital stay, regardless of the left ventricular (LV) dysfunction [29]. TAPSE represents a simple, and non-invasive method for assessing the RV systolic function [29]. The main cause of RV dysfunction in patients with HF is the pulmonary hypertension (PH), where several previous studies have reported the correlation between PH and mortality [29–31]. Naseem et al. [29] and Palazzuoli et al. [32] found that PASP, TAPSE, and TAPSE/PASP ratio were predictors for in-hospital mortality in HF patients. Also, Choudhary et al. [30] and Bursi et al. [31] found that elevated PASP could predict re-admissions and strongly predicts death among HF patients. Furthermore, RVOT1 was positively correlated with outcome and increased adverse events in patients with AHF. Palazzuoli et al. [32] found that RVOT1 > 40 mm related to 6 months outcome in AHF patients.

Here, we reported significant differences of TAPSE, SPAP, and RVOT1 between HF groups. SPAP and ROVT showed positive correlation with hospital stays, meanwhile, TAPSE showed a negative correlation. Although, these markers did not correlate with in-hospital death. SPAP was higher in congested groups (Warm-wet and Cold-wet groups), meanwhile, the isolated RHF and Cold-wet groups showed the highest levels of RVOT1 and the lowest level of TAPSE.

Additionally, LV enlargement in HF patients with reduced LV ejection fraction (HFrEF) showed a powerful predictor of adverse outcomes [33, 34]. Ito et al. [34] found that both LV volumes and diameters were independently associated with increased the adverse outcomes in HFrEF patients including all-cause death, CV death and events, HF hospitalization, and myocardial infarction (MI). Also, Narayanan et al. [33] found that LV diameter associated with increase of sudden cardiac death independent of the LVEF.

A few reports have been published on the effect of ventricular remodeling, such as interventricular septum thickness, with controversial results [35]. Li et al. [35] found that IVSD did not associated with death in dilated cardiomyopathy patients. Also, Biton et al. [36] found that patients with low relative wall thickness (eccentric hypertrophy) had increased risk for ventricular tachyarrhythmias (VAs) and increased in VAs risk or death.

The current study found that LVIDd and IVSD showed differences between HF groups, however, only LVIDd showed positive correlation with in-hospital stay but not with mortality. These previous echocardiographic features; including TAPSE, SPAP, RVOT1, IVSD, and LVIDd; did not show correlations with in-hospital mortality, which could due to small sample of our study.

Congestion, either pulmonary or systemic, is the main cause for hospitalization in HF patients, as a consequence of increased cardiac filling pressures [37]. While physical examination of pulmonary congestion has a low sensitivity and specificity, especially the lung auscultation is poorly correlated with LV filling pressures [37]. LUS has gained popularity as a quick examination to assist the pulmonary congestion with high sensitivity and specificity, and the cardiology community has extended its use to diagnose and manage congestion in patients with HF [9]. Also, echocardiography has the ability to non-invasively estimate LV filling pressures and assessing congestion in HF patients [37, 38].

E/e’ ratio is widely used and values ≥ 14 is correlated with invasive measurements of LV filling pressures [37]. E/e’ ratio has a good correlation with LV filling pressures in HF patients with preserved EF (HFpEF), meanwhile, there is only moderately correlation with LV filling pressures when E/e’ ratio > 15 in patients with HFrEF [37]. Mullens et al. [39] found that sensitivity (66%) and specificity (50%) for mitral E/e’ ratio > 15 to identify a pulmonary capillary wedge pressure (PCWP) > 18 mmHg and concluded that E/e’ ratio might not be a reliable in predicting LV filling pressures in advanced decompensated HF patients. Dini et al. [40] found that E/e′ ratio ≥ 13 or DT < 150 ms were closely associated with elevated LV filling pressure with a sensitivity of 87% and a specificity of 90% in detecting patients with elevated PCWP [40]. Moreover, DT can be unreliable marker for detecting LV filling pressure in patients with HFpEF, in which DT can be normal despite elevated LV filling pressures [16]. On the other hand, in patients with moderate to severe mitral annular calcification, IVRT (< 60 ms) was indicative for elevated LA pressure [40], however, IVRT is affected by tachycardia and arterial pressure [41].

Here, we found significant differences in DT, IVRT, and E/e’ ratio between HF groups. E/e’ Average was higher in the cold-wet group, while IVRT and DT were lower in the cold-wet group. With respect to pulmonary congestion, the previous studies put the cut-off values of E/e’ ratio, IVRT, and DT based on LV filling pressures guided by invasive procedures (such as PCWP). In our study, the cut-off values of E/e’ ratio, IVRT, and DT were (12.5, 55ms, and 131.5 ms; respectively), which showed lower values than previous studies in detecting congestion. On the other hand, the ESC 2021 reported the E/e’ ratio > 9 had a sensitivity of 78%, a specificity of 59% for the presence of HFpEF by invasive exercise testing, meanwhile, a higher cut-off of 13 had lower sensitivity (46%) but higher specificity (86%) [6]. Our study showed different values of these measures, which might result from the different and small populations, and LUS-guided for classifying the congestion. Also, the cut-off values in previous studies guided by invasive procedures might give a more precise value than this study, and these cut-off values differ between studies based on patients’ status (such as acute or chronic HF, HFpEF, or HFrEF).

These echocardiographic measures (E/e’ ratio, IVRT, and DT) were guided by LUS and showed good sensitivities of (96%, 74%, and 62%; respectively) and specificities of (53%, 92%, and 84%; respectively) in detecting congestion. Of note, the elevated LV filling pressures may precede the occurrence of clinical congestion by days or weeks [37], this was observed in our patients with warm-dry group. So, interpreting the LUS aligned with echocardiographic measures (E/e’ ratio, IVRT, and DT) seemed to be of great benefit in detecting congestion more than clinical assessment and easier than invasive procedures for guide the HF management.

This study has several limitations. The main limitation is the limited number of patients included from two university hospitals, especially the small number of cold-dray group. Also, no follow-up after patients were discharged and biomarkers such as NT-pro BNP were not included in this study because of limited resources in our hospitals. Despite these limitations, this study included all HF phenotypes including isolated right HF group and left HF groups. Also, LUS aligned with physical examination was used to accurately detect the congestion and guide the classification of the HF patients. Furthermore, several echocardiographic measures were included and analyzed between HF groups in addition to studying their effects on outcomes.

## Conclusion

Classifying AHF patients into these five groups, including isolated right HF and four groups of left HF, based on clinical examination supporting by echocardiography and LUS evaluation can give better assessment of the AHF phenotypes and gives more details for management. Aligned with ESC phenotypes, the isolated RHF in this classification should be included in future studies for management and follow-up for AHF patients. The worst outcomes in cold-wet and isolated RHF patients supports the fact that hypoperfusion as a marker of mortality and severity of HF, which needs special attention and management. The echocardiographic features showed correlations with in-hospital stays but not with mortality, however, this needed future large studies with long term of follow-up. The bedside diagnostic assessment by LUS and echocardiography is an easy tool and seems to be of great benefit in detecting congestion with good sensitivity and specificity that enhances the treatment protocols.

## Data Availability

All necessary details are available in the article. Further inquiries can be directed to the corresponding author.
